# Acquired thrombotic thrombocytopenic purpura with possible association with AstraZeneca‐Oxford COVID‐19 vaccine

**DOI:** 10.1002/jha2.219

**Published:** 2021-05-18

**Authors:** Mona Al‐Ahmad, Mona Al‐Rasheed, Neveen Abo Bakr Shalaby

**Affiliations:** ^1^ Microbiology Department Faculty of Medicine Kuwait University Kuwait Kuwait; ^2^ Al Rashed Allergy Centre Ministry of Health Kuwait; ^3^ Hematology Unit Department of Medicine Al Adnan Hospital Ministry of Health Kuwait

**Keywords:** covid‐19, thrombocytopenia, TTP, vaccines

## Abstract

Acquired thrombotic thrombocytopenic purpura is characterized by the microvascular aggregation of platelets and microangiopathic hemolytic anemia causing ischemia of multiple organs including the brain mainly and less likely the kidney and the heart. The disease is caused by severe reduction in the activity of ADAMTS 13 due to presence of inhibitory antibodies.

We report a case of a 37‐year‐old man with acquired thrombotic thrombocytopenic purpura (aTTP) that developed after AstraZeneca‐Oxford COVID ‐19 vaccination. He presented with progressive dizziness, fatigue, and headache associated with exertional shortness of breath and palpitation 10‐15 days after vaccination. Laboratory investigation showed hemolytic anemia, thrombocytopenia, and blood smear showed fragmented erythrocytes. aTTP was diagnosed based on the clinical presentation and was confirmed later by low level of ADAMTS13 activity and the presence of inhibitory antibodies. He was successfully treated using eight sessions of plasma exchange, corticosteroids, and rituximab.

To our knowledge, this case appears to represent the first report of aTTP following coronavirus disease 2019 (COVID‐19) vaccination.

## 1 CASE REPORT

A 37‐year‐old Kuwaiti gentleman presented to the emergency department of a general hospital complaining of a gradually progressive dizziness, fatigue, and headache associated with exertional shortness of breath and palpitation for 1 week, and he also noticed dark urine and red spots over his extremities 1 day prior to admission.

The patient is a heavy smoker who is complicated by secondary polycythemia for which he donates blood once every year.

He had venesection done a month prior to his presentation. The complete blood count (CBC) at the time of venesection showed hemoglobin of 17 g/L and normal platelet count.

Three weeks before his current admission, he received the first dose of AstraZeneca‐Oxford COVID‐19 vaccine, and his symptoms started 10 days to 2 weeks after receiving the vaccine.

On examination, the patient was alert and oriented. His blood pressure, temperature, and O_2_ saturation were within normal; however, he was tachycardic. The general examination revealed pallor, jaundice, and scattered purpuric eruptions on his extremities. Systemic examination for the cardiovascular, abdominal, and respiratory system was normal. His neurological examination was normal with no focal neurological deficit

## 2 INVESTIGATIONS

Initial complete blood count demonstrated the following: hemoglobin of 83 g/L with 8% reticulocytes, a low platelet count of 14 × 10^9^/L, and normal white blood cells. His blood smear demonstrated polychromasia, and 14% fragmented red blood cells. Hemolytic workup was positive, with high lactate dehydrogenase (LDH) of 1138 IU/L, a low haptoglobin of less than 0.1 g/L, indirect hyperbilirubinemia, and negative coombs test (Table 1).

**TABLE 1:** Blood investigation on admission and discharge and follow‐up

		Reticulocyte	Platelet	LDH
Date/ investigation	Hg g/L	%	×10^9^/L	IU/L
On admission	83	8%	14	1138
On discharge	123	2%	340	199
One month after discharge	141	2%	304	175

Coagulation profile including prothrombin time, partial thromboplastin time, fibrinogen, and D dimer was normal. Renal and liver functions were normal apart from indirect hyperbilirubinemia. Troponin level was normal. The patient was tested negative to SARS‐CoV‐2 virus by polymerase chain reaction test.

ADAMTS13 activity was 2.6% and antibodies assay was positive (these tests were sent before the start of plasma exchange and the results were received with 3 days). Chest X‐ray was done with no obvious pathology.

## 3 DIAGNOSIS

Patient was found to have thrombocytopenia and hemolytic anemia with picture of microangiopathic hemolytic anemia (MAHA) in blood smear, so he was diagnosed as aTTP after exclusion of other TTP‐like conditions such as disseminated intravascular coagulation (DIC), malignant hypertension, or hemolytic uremic syndrome (HUS). This diagnosis was confirmed later with low ADAMTS 13 level and positive inhibitory antibodies. The patient was investigated for possible causes of secondary aTTP such as autoimmune diseases and HIV infection and the result of the investigation were all negative.

## 4 TREATMENT AND OUTCOME

Plasma exchange was started immediately planned for daily exchange with 1.5 plasma volume. Methylprednisolone at a dose of 1 g intravenous was started for 3 days initially and was followed by prednisolone 1 mg/kg/day. Rituximab 375 mg/m^2^ was added as once weekly infusion for 4 weeks.

The patient received total of eight sessions of plasma exchange while in hospital. The patient symptomatology has improved, and his blood investigations were back to normal with LDH of 199 IU/L, platelet count of 340 × 10^9^/L, and Hg level of 123 g/L on discharge.

The patient had completed the remaining of the four doses of rituximab as an outpatient and he had a rapid taper off the steroid dose over 3 weeks.

## 5 DISCUSSION

aTTP is a rare disease with an incidence of 3 to 10 cases per million adult per year [1]. It is caused by severe reduction in the activity of Von Willebrand factor cleaving metalloprotease (ADAMTS13). This is, in turn, caused by the presence of inhibitory antibodies.

The disease is characterized by small vessel platelet‐rich microthrombi that results in MAHA, thrombocytopenia, and thrombosis.

The historical pentad of fever, thrombocytopenia, microangiopathic hemolytic anemia, neurological symptoms, and renal insufficiency appears obsolete, as these symptoms were present in <10% of patients with aTTP [2].

The symptoms of the disease initially include fatigue, shortness of breath, abdominal pain, nausea, vomiting, and petechial rash or minor bleeding.

As the disease progresses and becomes more aggressive, the disease can affect mainly the central nervous system (CNS) and the patient may developed any neurological symptoms including coma, strokes, seizure, TIA, headache, or confusion. Other organs that can be affected are the gastrointestinal tract, kidneys, heart, and rarely the pulmonary system [2, 3].

aTTP can be primary or it can be associated with other conditions. The most common clinical conditions associated with aTTP are bacterial infections and autoimmune diseases like systemic lupus erythematous, in addition to the antiphospholipid syndrome, pregnancy, HIV infection, pancreatitis, cancers, organ transplantation, and drugs including mitomycin C, cyclosporine, quinine, clopidogrel, and ticlopidine [4, 5, 6].

Laboratory investigation reveals MAHA [7], thrombocytopenia (typically <30 × 10^9^/L), normal to mild renal impairment, increased cardiac troponin level (>0.1 μg/L) with no clinical cardiac involvement [8]. Electrocardiogram changes such as repolarization, elevated serum lactate dehydrogenase, elevated tota and indirect bilirubin, reduced to absent serum haptoglobin, normal coagulation screen, normal direct antiglobulin test (DAT), and reduced ADAMTS13 level (below 10%) with the presence of inhibitory antibodies [2].

PLASMIC score is a point‐based prediction scores that have been validated to predict an acquired ADAMTS13 deficiency and aTTP. The scores include platelet count of <30 × 10^9^/L, indicator of hemolysis, absence of active cancer, absence of solid organ or hematopoietic stem cell transplant, serum creatinine level < 177 mcmol/L, mean corpuscular volume (MVC) < 90 fL, and international normalised ratio (INR) < 1.5 [9].

aTTP requires urgent management, in intensive care units, as a medical emergency. The mainstay of the treatment is therabutic plasma exchange (TPE) that is done for all patient suspected to have aTTP because the disease can be fatal if not treated early [10, 11]. Plasma infusion can be given as a temporary measure for patient with an expected delay in initiation TPE. The addition of immunosuppressive agent including corticosteroid and rituximab has improved the outcome of the aTTP and it has decreased the duration of TPE. These agents are given to patient with intermediate to high PLASMIC score [12].

Vaccination in general is an important part of preventative medicine and COVID‐19 vaccination is proven to reduce morbidity and mortality of the disease.

Rarely vaccination, especially against viral infection, has been associated with autoimmune pathology including aTTP. Three case reports have linked influenza vaccine with aTTP [13, 14, 15]: one case report has linked pneumococcal vaccination with aTTP [16], one case report has described aTTP after H1N1 vaccination [17], and one case report of aTTP shortly after Rabies vaccination [18].

To our knowledge, this case report is the only case showing a possible connection of AstraZeneca‐Oxford COVID‐19 vaccination to aTTP. The patient had no prior history of any form of thrombocytopenia or other hematological disorder, and no medication apart from the vaccination. Screening for secondary causes of aTTP was all negative.

## 6 CONCLUSION

This report suggests the potential, but not proven role, of AstraZeneca‐Oxford COVID‐19 vaccination in the pathogenesis of aTTP. Vaccine‐related aTTP, like other forms of aTTP, can be successfully treated by plasmapheresis, corticosteroids, and rituximab.

## CONFLICT OF INTEREST

The authors have no conflicts of interest to declare

## AUTHORS CONTRIBUTION

MA and EJ initiated and coordinated the development of the paper and worked on data collection, analysis, and writing up the paper. NA and JN analyzed and interpreted the results and helped in writing up introduction. NA, JN, and EJ were major contributors in writing up the manuscript. All authors read and approved the final manuscript.
